# Genetically Modified Human Bone Marrow Derived Mesenchymal Stem Cells for Improving the Outcome of Human Islet Transplantation

**DOI:** 10.1371/journal.pone.0077591

**Published:** 2013-10-29

**Authors:** Vaibhav Mundra, Hao Wu, Ram I. Mahato

**Affiliations:** Department of Pharmaceutical Sciences, University of Tennessee Health Science Center, Memphis, Tennessee, United States of America; Rutgers - New Jersey Medical School, United States of America

## Abstract

The objective of this study was to determine the potential of human bone marrow derived mesenchymal stem cells (hBMSCs) as gene carriers for improving the outcome of human islet transplantation. hBMSCs were characterized for the expression of phenotypic markers and transduced with Adv-hVEGF-hIL-1Ra to overexpress human vascular endothelial growth factor (hVEGF) and human interleukin-1 receptor antagonist (hIL-1Ra). Human islets were co-cultured with hBMSCs overexpressing hVEGF and hIL-1Ra. Islet viability was determined by membrane fluorescent method and glucose stimulation test. Transduced hBMSCs and human islets were co-transplanted under the kidney capsule of NOD.Cg-*Prkdc^scid^ Il2rg^tm1Wjl^*/SzJ (NSG) diabetic mice and blood glucose levels were measured over time to demonstrate the efficacy of genetically modified hBMSCs. At the end of study, immunofluorescent staining of kidney section bearing islets was performed for insulin and von Willebrand Factor (vWF). hBMSCs were positive for the expression of CD73, CD90, CD105, CD146 and Stro-1 surface markers as determined by flow cytometry. Transduction of hBMSCs with adenovirus did not affect their stemness and differentiation potential as confirmed by mRNA levels of stem cell markers and adipogenic differentiation of transduced hBMSCs. hBMSCs were efficiently transduced with Adv-hVEGF-hIL-1Ra to overexpress hVEGF and hIL-1Ra. Live dead cell staining and glucose stimulation test have shown that transduced hBMSCs improved the viability of islets against cytokine cocktail. Co-transplantation of human islets with genetically modified hBMSCs improved the glycemic control of diabetic NSG mice as determined by mean blood glucose levels and intraperitoneal glucose tolerance test. Immunofluorescent staining of kidney sections was positive for human insulin and vWF. In conclusion, our results have demonstrated that hBMSCs may be used as gene carriers and nursing cells to improve the outcome of islet transplantation.

## Introduction

Type I diabetes is an autoimmune disease characterized by the destruction of islets. Transplantation of human islets has been clinically used to achieve insulin independence. However, in addition to long-term graft rejection, clinical success of islet transplantation is limited by primary non function (PNF) of islets [1]. PNF is characterized by poor glycemic response due to β-cell death in the first two weeks after transplantation [2]. There is marked β-cell death immediately after transplantation due to hypoxia and exposure to inflammatory cytokines released by infiltrating immune cells [3]. Human islets upon exposure to inflammatory cytokines show increased expression of apoptosis markers [4]. Interleukin-1 beta (IL-1β) produced by islets and macrophages plays a key role in the apoptosis of islets after transplantation [5]. hIL-1Ra competitively blocks IL-1β binding and has been shown to minimize the detrimental effect of IL-1β on islet viability [5], [6].

Islets in pancreas have dense network of capillaries, much higher as compared to surrounding exocrine tissues [7], [8]. Besides rich blood supply, there is an endothelial endocrine axis within adult pancreas, which is essential for the proliferation of β cells [9]. Islet isolation and purification process not only destroys external vasculature but also adversely affects the intra islet endothelial cells [10]. Revascularization of islet grafts is an essential event for the success of islet transplantation [11]. It has been demonstrated that vascular endothelial growth factor (VEGF) stimulates angiogenic capacity of intra islet endothelial cells and also recruits recipient endothelial cells to form new microvessels [12], [13].

We previously demonstrated that bipartite adenoviral vector encoding hVEGF and hIL-1Ra can improve the viability and revascularization of transplanted islets [14]. However, adenovirus transduction of islets results in immune response especially when relatively higher multiplicity of infection (MOI) is used to get desired transgene expression [5], [15]. To resolve this potential issue, we have used human bone marrow mesenchymal stem cells (hBMSCs) as gene delivery vehicles. In addition, hBMSCs also provide immune and anti-inflammatory protection to islet grafts [16].

In this report, we have shown that hBMSCs were transduced with bipartite adenoviral vector to coerexpress hVEGF and hIL-1Ra. The effect of Adv-hVEGF-hIL-1Ra transduced hBMSCs on islet viability and glucose stimulation was determined. Islets were co-transplanted with transduced hBMSCs under the kidney capsule of diabetic NSG mice to determine the effect of stem cells and gene therapy on the glycemic control and revascularization of transplanted islets.

## Materials and Methods

### Materials

Replication-deficient (ΔE1/ΔE3) Adv-hVEGF-hIL-1Ra containing a cytomegalovirus (CMV) promoter, hVEGF cDNA, and rabbit β-globin poly A in the E-1 region, and a CMV promoter, hIL-1Ra cDNA, and rabbit β-globin poly A in the E-3 region was constructed amplified, and the titer was measured in our laboratory as previously described [17]. Primary hBMSCs, HyClone Advanced Stem Cell Medium and Medium Supplement were purchased from Thermo Fisher Scientific (Waltham, MA). Human islets were received from Integrated Islet Distribution Program (Duarte, CA). CMRL-1066 medium for islet culture, propidium iodide (PI) and 6-diamidino-2-phenylindole (DAPI) were purchased from Sigma Aldrich (St. Louis, MO). Fetal bovine serum (FBS) was purchased from MediaTech Cellgro (Herndon, VA). Phosphate-buffered saline (PBS) was purchased from GIBCO-BRL (Gaithersburg, MD). hVEGF and hIL-1Ra ELISA kits and recombinant inflammatory cytokines (IL-1β, TNF-α and IFN-γ) were purchased from R&D Systems (Minneapolis, MN). Human insulin and c-peptide ELISA kits were purchased from Alpco Diagnostics (Windham, NH). The primary antibodies for human insulin, human von Willebrand Factor (vWF), and the Dylight 488-conjugated secondary antibody were purchased from Abcam (Cambridge, MA). Alexa Fluor 568- conjugated secondary antibody and 0.25% trypsin were purchased from Invitrogen (Carlsbad, CA). Eight-well Lab Tek Chamber slides were purchased from Nalge Nunc. (Rochester, NY). Ultrasensitive One Touch glucose test strips and One Touch Ultra glucometer were purchased from LifeScan (Milpitas, CA). Tissue-Tek optimal cutting temperature (O.C.T.) compounds were purchased from Sakura Finetek (Torrance, CA).

### Characterization, Transduction and Differentiation of hBMSCs

Fluorochrome-coupled antibodies specific for Stro-1, CD73, CD90, CD105 and CD146 were used to analyze surface markers. Flow cytometric data were acquired using LSR II Special Option Flow Cytometer (BD Biosciences) and analyzed using FACSDiva software (BD Biosciences).

hBMSCs were plated at a density of 0.2 million cells in 6 well plates for 24 h and transduced with Adv-hVEGF-hIL-1Ra for 3 h at 100 and 200 MOI. hBMSCs were then washed thrice with PBS and subsequently cultured for 10 days. Cell culture media was collected every two days to estimate hVEGF and hIL-1Ra gene expression by ELISA. Total cellular RNA was isolated using an RNeasy mini kit (Qiagen, Valencia, CA) and the extracted RNA (1 µg) was reverse transcribed to generate the first strand cDNA. Two microliters of cDNA were used as a template and analyzed by SYBR Green-I dye universal PCR master mix on a LightCycler 480 Instrument. To assess the specificity of the amplified PCR product, melting curve analysis was performed on a LightCycler 480 Instrument. The results of stem cell markers (Nanog and Oct4) mRNA levels were compared by calculating the cross point value and normalized for GAPDH reference gene.

To determine the effect of Adv-hVEGF-hIL-1Ra transduction on hBMSC differentiation, hBMSCs were cultured in MEM alpha media containing 10% FBS, 1% antibiotics (streptomycin+penicillin), 1 µM dexamethasone, 0.5 mM 3-isobutyl- 1-methylxanthine, 0.2 mM indomethacin and 10 µg/ml insulin after transduction at 200 MOI for 3 h. Adipogenic differentiation media was replaced every 3–4 days for two weeks. Adipogenic differentiation of hBMSCs was assessed using Oil Red staining. For osteogenic differentiation, transduced hBMSCs were incubated at 50–70 percent confluence in MEM alpha media containing 10 M dexamethasone, 0.2 mM ascorbic acid mM and β-glycerolphosphate. The medium was replaced every 3–4 days for 21 days before fixing and Alizarin Red S staining.

### Islet Viability Study

After transduction with Adv-hVEGF-hIL-1Ra at 200 MOI for 3 h, 5×10^5^ hBMSCs were trypsinized (0.25%) and co-cultured with 500 IEs in a 10 cm dish of CMRL-1066 medium with 10% FBS for 2 days. The islet/hBMSC co-culture was stimulated with cytokine cocktail (5 ng/ml IL-1β, 10 ng/ml TNF-α and 50 ng/ml IFN-γ) for 10 days and stained with 5 µg/ml calcein-AM and 2 µg/ml propidium iodide for 30 min to assess islet viability under a fluorescent microscope.

Insulin production of human islets was quantified by the static insulin release method. Briefly, 500 IEs were cultured alone or with 5×10^5^ untransduced or with 5×10^5^ Adv-hVEGF-IL-1Ra transduced hBMSCs (200 MOI) in a 10 cm dish containing CMRL-1066 medium with 10% FBS for 10 days. Cytokine cocktail was added on day 2 and the islet/hBMSC co-culture was incubated with cytokine cocktail for 8 days. Insulin release from islets was measured at day 10 by sequentially stimulating islets with Kreb’s buffer containing 2.5 mM (basal) and 22 mM glucose (stimulated) at 37°C for 1 h.

### Islet Transplantation

Animal experiments were performed in accordance with the NIH guidelines using a protocol approved by the Institutional Animal Care and Use Committee (IACUC) of the University of Tennessee Health Science Center. Diabetes was induced by intraperitoneal injection of low dose streptozotocin (STZ) (70 mg/kg) to NSG mice for 2 consecutive days. Animals were classified as diabetic when two consecutive measurements of blood glucose were ≥400 mg/dl with a glucometer. Prior to receiving human islets from the Integrated Islet Distribution Centers, hBMSCs were transduced with Adv-hVEGF-hIL-1Ra at 200 MOI. Islets and hBMSCs were co-transplanted under the kidney capsules of STZ induced diabetic mice. Three shots of insulin (5 U/kg) were given to each mouse on the first 3 days after transplantation to relieve the hyperglycemic stress to the newly-transplanted islets [18]. The non-fasted glucose levels of all the mice were measured from the snipped tail of each animal at 14.00 h during the first week after surgery and then weekly. At the end of study, the mice were anaesthetized by isoflurane inhalation to collect blood to measure human serum insulin and c-peptide levels by ELISA.

### Intraperitoneal Glucose Tolerance Test

Fifteen days after islet transplantation, glucose tolerance was determined in overnight-fasted mice, which were subjected to intraperitoneal injection of glucose at 2 g/kg of body weight [19]. Blood samples were obtained from the snipped tail at 15, 30, 60, 90 and 120 min after injection and analyzed for glucose levels with a glucometer.

### Immunofluorescence Staining

Mice from each group were sacrificed at the end of study and the kidneys bearing islets were removed, washed with PBS, fixed in 4% paraformaldehyde overnight, and embedded in optimal cutting temperature (O.C.T.). Frozen sections of 10 mm thickness were cut. To detect insulin positive human islets, the slides were stained with guinea pig anti-human insulin primary antibody (dilution 1∶200) at 4°C overnight and Alexa Fluor 568-conjugated goat anti-guinea pig secondary antibody (dilution 1∶500) at room temperature for 1 h. To detect revascularization, the slides were stained with rabbit anti-human vWF primary antibody (dilution 1∶500) at 4°C overnight and Dylight 488-conjugated goat anti-rabbit secondary antibody (dilution 1∶500) at room temperature for 1 h. Slides were counter-stained with 4′, 6-diamidino-2-phenylidone (DAPI).

### Statistical Analysis

Statistical significance of the difference between the two groups was determined by an unpaired Student’s t-test and between several groups by one-way analysis of variance.

## Results

### Characterization, Transduction and Differentiation of hBMSCs

**Figure 1:**
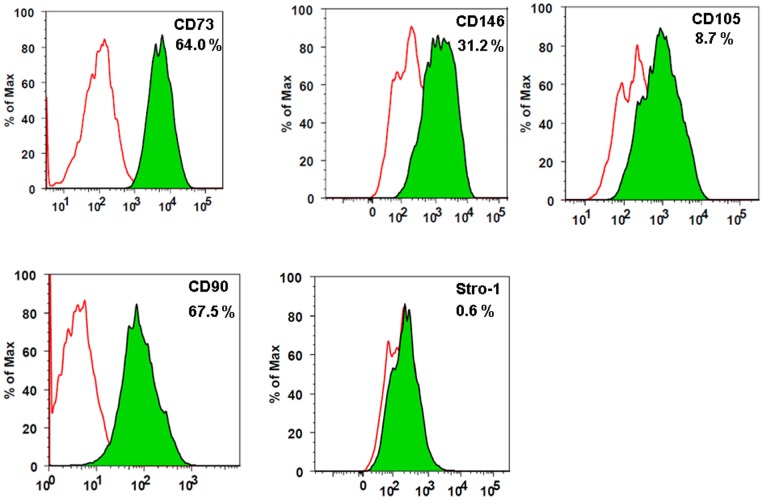
Phenotypic characterization of cultured hBMSCs. hBMSCs are positive for CD73, CD146, CD105, CD90 and Stro1. Open peaks indicate the isotype of each cell and solid peaks represent expression of each marker. Numbers in the box specify the percentage of positive cells as compared to isotype control.

**Figure 2:**
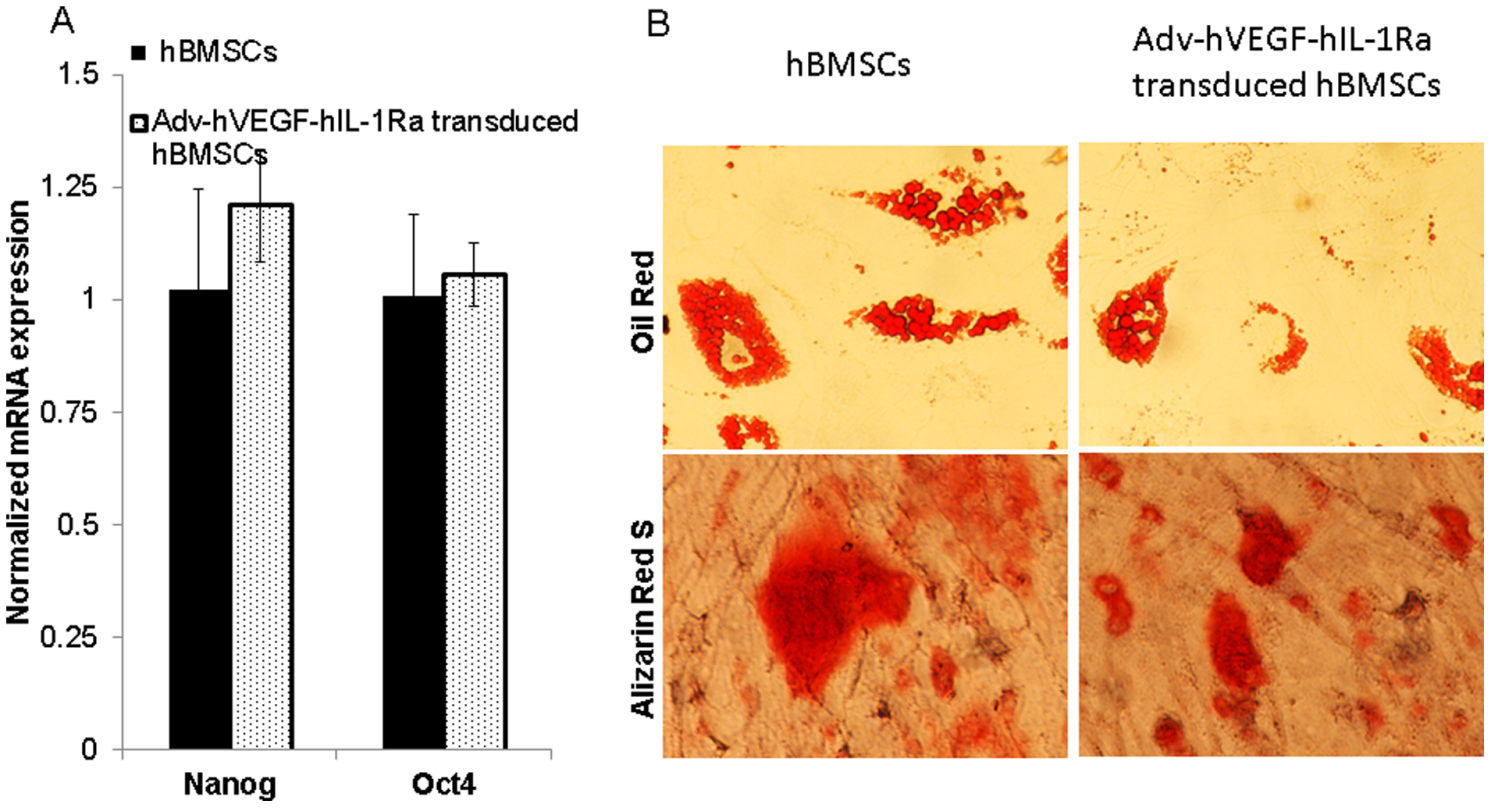
Expression of stem cell markers and differentiation of hBMSCs after transduction with Adv-hVEGF-hIL-1Ra at 200 MOI. (A) Real Time RT-PCR analysis to quantify change in the expression of Nanog and Oct4 stemness markers. Data are represented as the mean ± SD, n = 3. (B) Oil Red and alizarin red S staining of hBMSCs after culture in adipogenic and osteogenic differentiation media, respectively.

Flow cytometric analysis showed hBMSCs were positive for cell surface markers CD73, CD146, CD105, CD90 and Stro-1 (Figure 1). Nanog, Oct4 and Sox 2 are known pluripotency markers and their expression indicates stem cell immaturity. Real Time RT-PCR data have shown that expression of these markers was not affected by the adenovirus transduction suggesting that hBMSCs can be used as gene delivery vehicles without affecting their stemness (Figure 2A).

**Figure 3:**
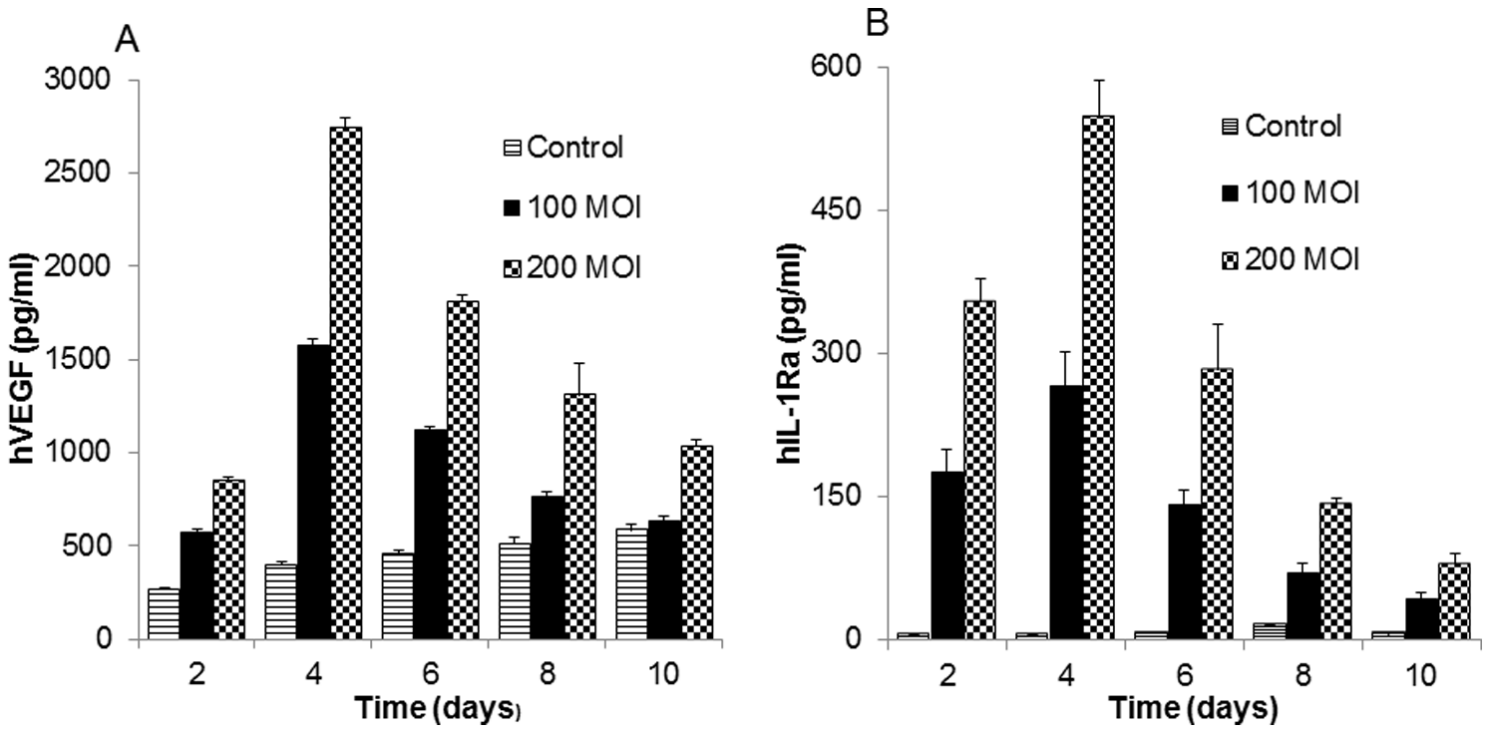
Expression of hVEGF and hIL-1Ra. hBMSCs were transduced with Adv-hVEGF-hIL-1Ra at 100 and 200 MOIs and cell culture medium was collected to perform ELISA. (A) hVEGF and (B) hIL-1Ra. Data are represented as the mean ± SD, n = 3.

Transduction of hBMSCs with Adv-hVEGF-hIL-1Ra resulted in significant overexpression of hVEGF and hIL-1Ra, which peaked at day 4 and gradually returned to basal levels at day 10 (Fig. 3A and 3B). We observed dose and time dependent expression of hVEGF and hIL-1Ra. Overexpression of transgenes is transient as adenoviral vector does not result in genomic integration.

Transduction of hBMSCs with adenovirus did not affect the differentiation potential of hBMSCs as evident from Oil Red staining of adipocytes from differentiated hBMSCs (Figure 2B)However, it did affect osteogenic differentiation which was evident from the extent of area of alizarin red positive cells.

### Islet Viability Study

**Figure 4:**
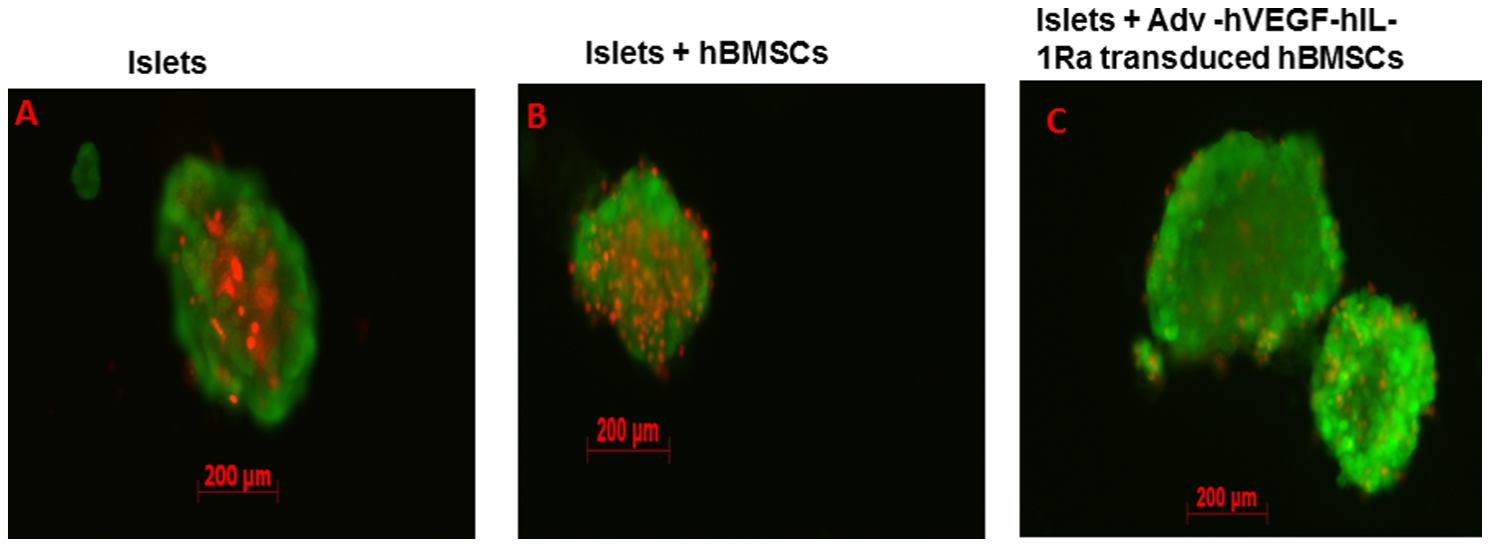
Protection of islets against cytokine cocktail as shown by Calcein AM and PI staining. (A) Islets, (B) Islets with hBMSCs, (C) Islets with hBMSCs transduced with Adv-hVEGF-hIL-1Ra at the ratio of 1 islet equivalent to 100 hBMSCs.

PNF of islets after transplantation is the primary reason for the loss of islet function. Inflammatory cytokine cocktail was used to simulate PNF. Prevention of PNF of islets by hBMSCs overexpressing hIL-1Ra was measured by the membrane fluorescent staining method. Islets upon exposure to inflammatory cytokines showed significant cell death as evident from red PI staining in the core of islets (Figure 4). While hBMSCs reduced the level of islet cell death, overexpression of hIL-1Ra by hBMSCs prevented the islet cell death as there are only few PI stained red cells in the islet (Figure 4).

**Figure 5:**
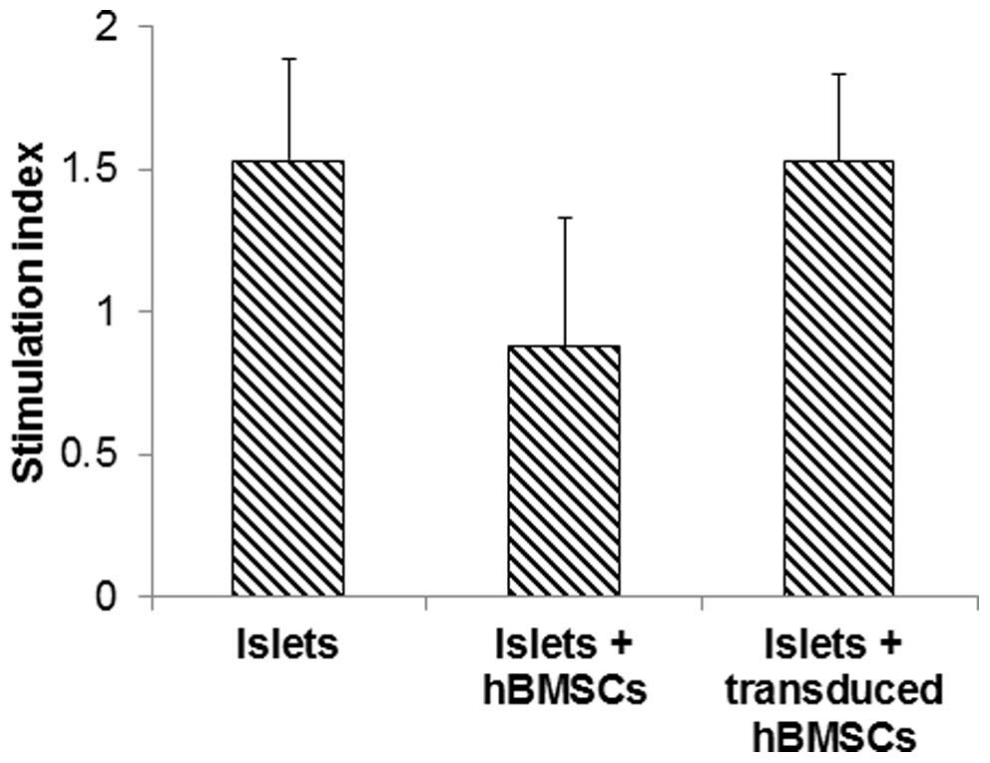
Stimulation index (SI) of human islets after incubation with inflammatory cytokine cocktail of 5 ng/ml IL-1β, 10 ng/ml TNF-α and 50 ng/ml IFN-γ for 10 days. Non-transduced islets were used as controls. SI was determined as the ratio of insulin released from islets when they were incubated in Kreb’s buffer containing 22 mM and 2.2 mM glucose. Data are presented as the mean ± SE (n = 3).

Islets alone upon culture for 10 days maintained insulin releasing function upon stimulation with glucose (SI = 1.52). Co-culture of hBMSCs alone with islets could not prevent the islet cell death, resulting in the stimulation index (SI) of insulin less than 1 (SI = 0.88) (Figure 5). However, overexpression of hIL-1Ra by hBMSCs restored insulin release by the islets in response to stimulatory glucose concentrations (SI = 1.53). Islet viability by membrane fluorescent staining and glucose stimulated insulin release assay have shown that overexpression of hIL-1Ra by hBMSCs could prevent the PNF and maintain insulin releasing function of islets to stimulatory glucose concentration.

### Cotransplantation of hBMSCs Overexpressing hVEGF and hIL-1Ra Improved Islet Transplantation

**Figure 6:**
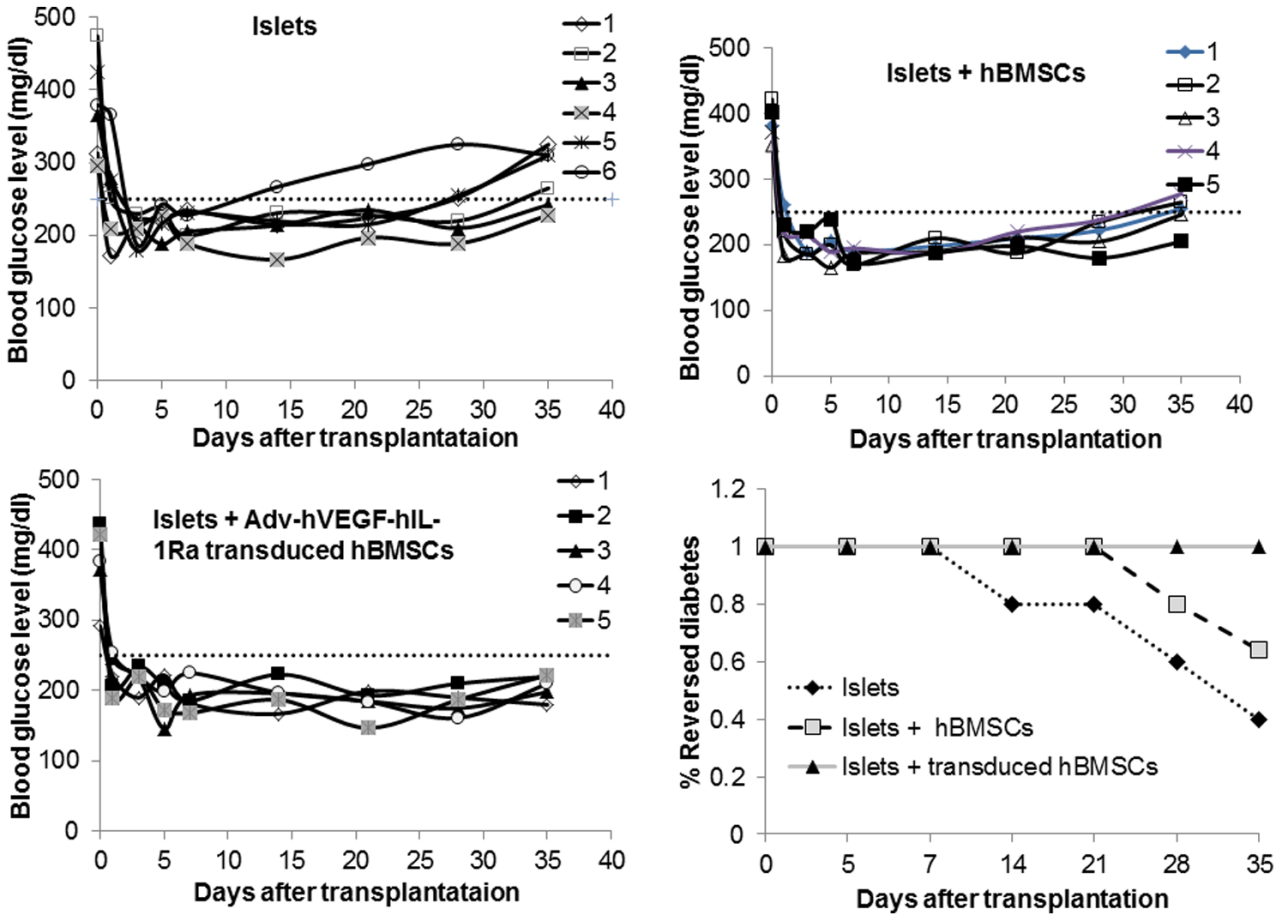
Effect of Adv-hVEGF-hIL-1Ra transduced hBMSCs on the outcome of human islet transplantation. (A, B, C) The blood glucose level of every mouse after receiving 2000 human islet equivalent transplanted either alone or hBMSCs before or after transduction with Adv-hVEGF-hIL-1Ra (A–C). (D) Diabetes reversal ratio of the diabetic mice after human islet transplantation. Blood glucose ≤250 mg/dl (dashed line) was identified as reversed-diabetes (dashed line in A, B, C). Black triangles indicate mice receiving islets with Adv-hVEGF-hIL-1Ra transduced hBMSCs; gray squares indicate mice receiving islets with hBMSCs; black diamond’s indicate mice receiving islets only.

**Figure 7:**
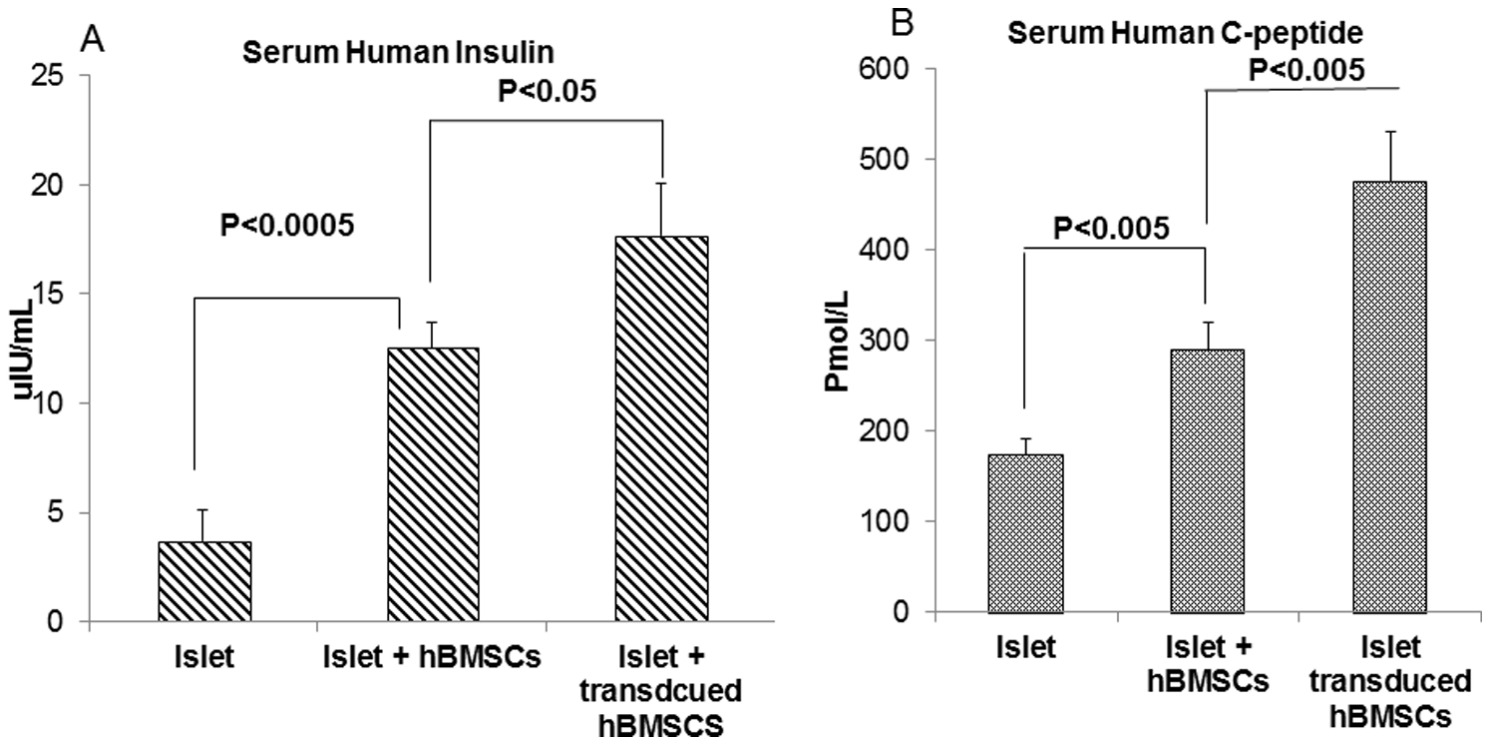
Effect of Adv-hVEGF-hIL-1Ra transduced hBMSCs on human serum insulin and C-peptide levels of diabetic NSG. Mice transplanted with either islet alone or with hBMSCs before or after transduction with Adv-hVEGF-hIL-1Ra at 35 days post transplantation (A) Human serum insulin (B) Human serum C-peptide. Data are presented as the mean ± SD, n = 5. p<0.05 under t-test.

Transplantation of 2000 islet equivalents (IEs) was able to reverse diabetes in all the transplanted animals. However, co-transplantation of islets with hBMSCs overexpressing hVEGF and hIL-1Ra resulted in better glycemic control in terms of the mean blood glucose level, duration of normoglycemia and response to glucose challenge. Islets alone could reverse diabetes only up to 21 days in most of the animals (Figure 6A). Although hBMSCs improved the outcome of islet transplantation, there was still rapid increase in blood glucose level when untransduced hBMSCs were utilized. In contrast, co-transplantation of islets with Adv-hVEGF-hIL-1Ra transduced hBMSCs significantly increased the diabetes reversal ratio, suggesting the positive role of Adv-hVEGF-hIL-1Ra transduced hBMSCs on islet transplantation (Figure 6D). To determine the functional viability of islet grafts after islet transplantation, the mice were anesthetized to collect blood to measure human serum insulin and C-peptide levels by ELISA. The levels of human insulin and C-peptide of the mice co-transplanted with Adv-hVEGF-hIL-1Ra-transduced hBMSCs were significantly higher than those of the mice transplanted with islets alone (Figure 7). We could not measure the blood glucose levels of NSG diabetic mice beyond 35 days post transplantation due to the closure of our laboratory at the University of Tennessee Health Science Center.

**Figure 8:**
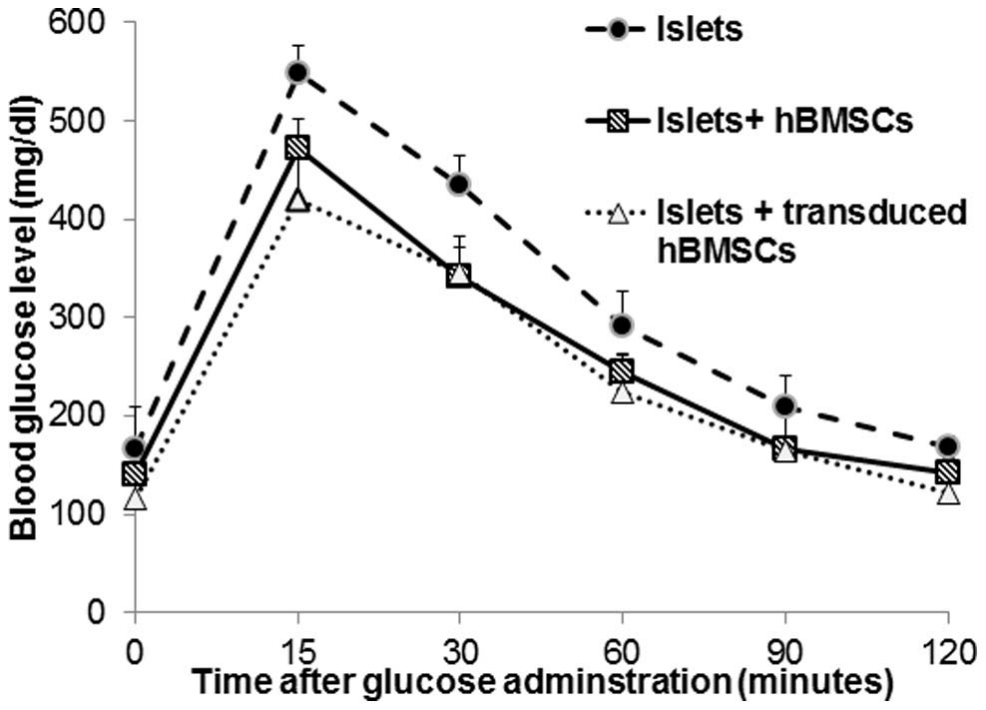
Intraperitoneal glucose tolerance test after 15 days of islet transplantation. Mice were fasted overnight and then injected intraperitoneally with glucose (2 g/kg of body weight). Blood glucose levels were measured by tail pricking at indicated time points with a glucometer. Data are presented as the mean ± SD (n = 5).

This improvement in islet transplantation was confirmed by performing intraperitoneal glucose challenge. Blood glucose levels increased immediately after glucose (2 g/kg) injection, peaked at 15 min in all groups, and then decreased over time. Co-transplantation of islets with transduced hBMSCs showed accelerated glucose clearance resulting in lower blood glucose levels at 15 and 30 min post glucose injection (Figure 8). Islets transplanted with Adv-hVEGF-hIL-1Ra transduced hBMSCs had a better engraftment outcome and led to superior glucose control and tolerance compared to islets or islets with hBMSCs.

### Immunofluorescence Staining

**Figure 9:**
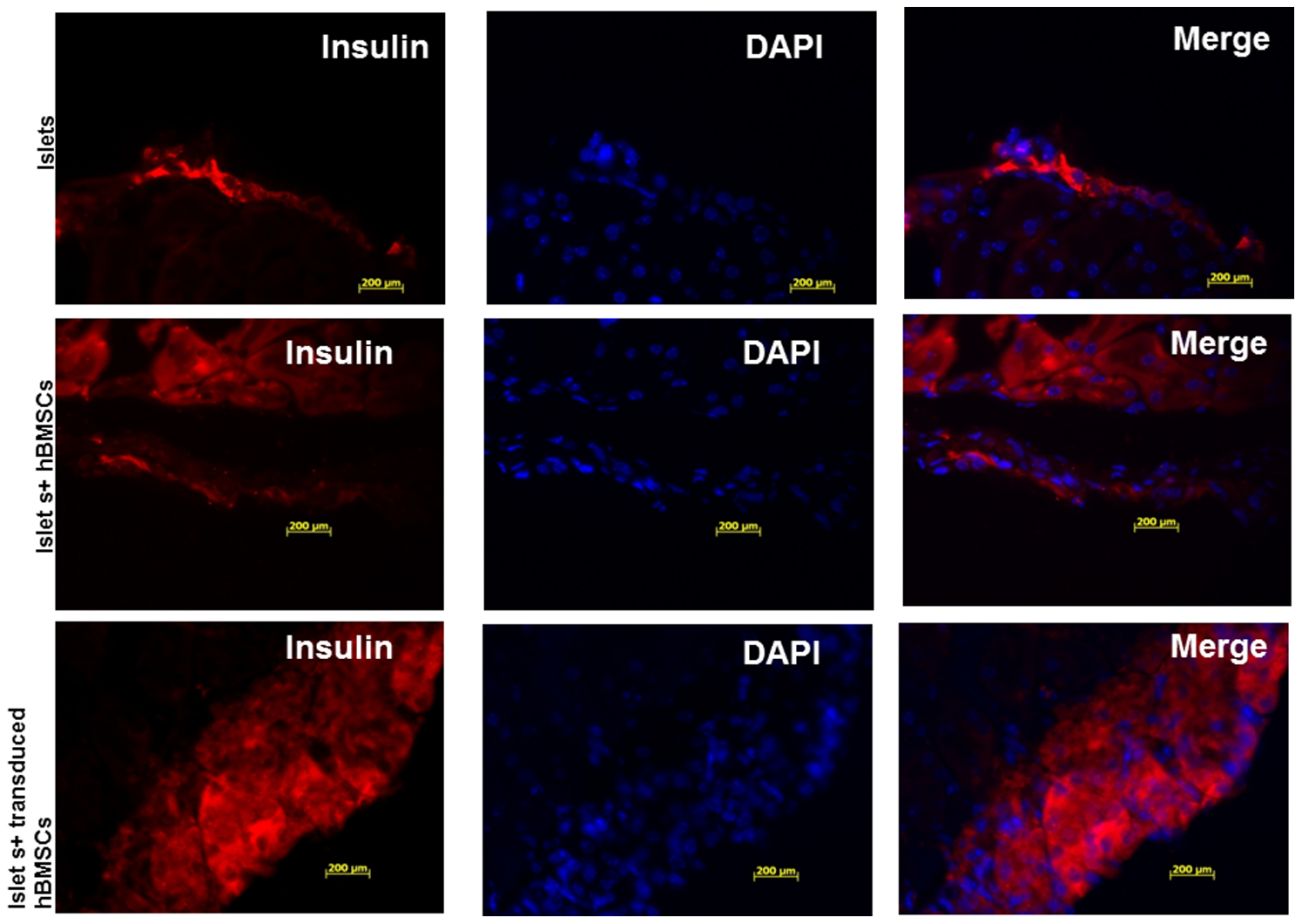
Immunofluorescence staining of the kidney section bearing human islets 35 days after islet transplantation. Insulin was stained in red to indicate the functional human islets after transplantation. Kidney section of mice bearing human islets; human islets co-transplanted with hBMSCs; human islets co-transplanted with Adv-hVEGF-hIL-1Ra transduced hBMSCs.

**Figure 10:**
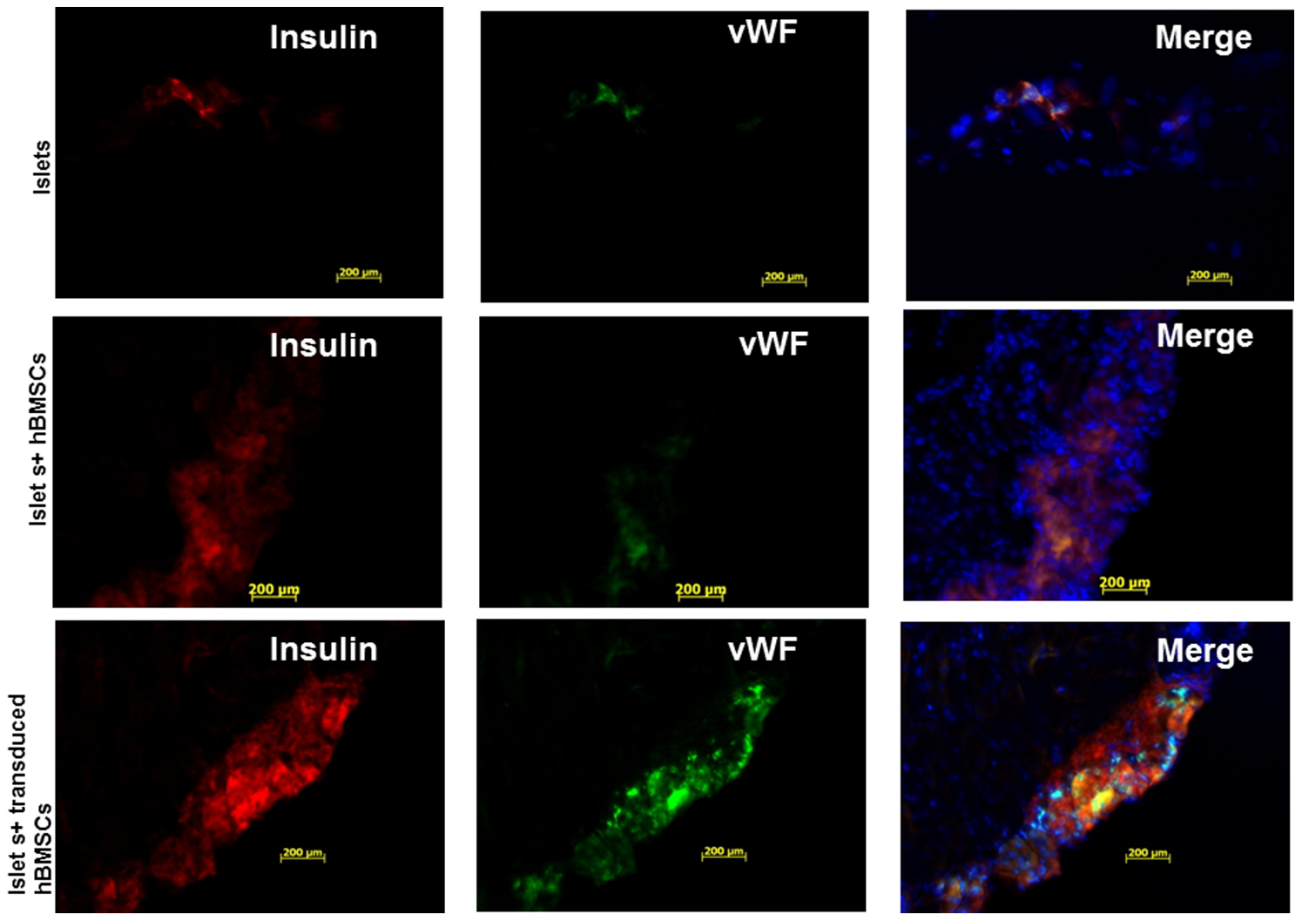
Immunofluorescence staining of the kidney sections bearing human islets 35 days after islet transplantation. Insulin was stained in red to indicate the functional human islets after transplantation. vWF was stained in green to indicate the revascularization of transplanted human islets. Kidney section of mice bearing human islets; human islets co-transplanted with hBMSCs; human islets co-transplanted with Adv-hVEGF-hIL-1Ra transduced hBMSCs.

Immunofluorescence staining of the kidney section bearing transplanted islets showed the engraftment of insulin-positive islets under the kidney capsule (Figure 9). vWF staining was performed to assess the extent of vascularization of islets. vWF staining was undetectable in the kidney sections bearing islets alone and sparse in the kidney sections bearing islets and un-transduced primary hBMSCs (Figure 10). However, vWF staining in the kidney sections bearing islets co-transplanted with Adv-hVEGF-hIL-1Ra transduced primary hBMSCs was detected. Analysis of human insulin and vWF immunofluorescence suggested increased revascularization of islets when co-transplanted with Adv-hVEGF-hIL-1Ra transduced primary hBMSCs (Figures 9 & 10).

## Discussion

Type I diabetes is characterized by autoimmune destruction of islets and requires lifelong administration of insulin to maintain normoglycemeia. Since the success of Edmonton protocol, islet transplantation has been performed in many patients especially undergoing kidney transplantation [20]. However, the proportion of recipients who achieved insulin independence is low and required supplemental administration of insulin to achieve normoglycemeia [21].

Majority of islets undergo apoptosis within a week or two after transplantation due to inflammatory reaction, mediated by IL-1β, TNF-α and IFN-γ released by infiltrating immune cells [22], [23]. Among various inflammatory cytokines, IL-1β is the major inflammatory cytokine whose expression is upregulated during islet isolation procedure, culture and upon exposure to hyperglycemic conditions [24]–[26]. Human islets exposed to IL-1β undergo NFkB activation, Fas upregulation and DNA fragmentation, eventually leading to β-cell death [27], [28]. To prevent IL-1β induced β-cell apoptosis, human islets have been genetically modified to overexpress hIL-1Ra locally [5]. Our group has also demonstrated that adenovirus mediated overexpression of hIL-1Ra can block IL-1β induced caspase activation, impaired glucose stimulated insulin release, and Fas triggered apoptosis [14], [17].

hBMSCs are heterogeneous population of cells and differ in clonogenicity, proliferation capacity and differentiation potential [29], [30]. The International Society for Cell Therapy (ISCT) has proposed expression of cell surface markers CD73, CD90 and CD105 as minimum criteria to characterize hBMSCs [31]. Stro-1 is a marker of early mesenchymal stromal precursor cells and strongly linked to hBMSCs colony forming efficiency, plasticity and other paracrine signaling [32], [33]. Level of expression of CD146 has been shown to correlate with the proliferation capacity of MSCs and their differentiation potential [30]. Our flow cytometry data (Figure 1) have shown that hBMSCs used in this study adhere to the minimum criteria set by ISCT and have high proliferation potential as evident by Stro-1 and CD146 expression.

hBMSCs can be readily transduced with all the known viral vectors and efficiently overexpress transgene [34], [35]. Ease of isolation, absence of immuno co-stimulatory receptors, their immunomodulatory (Figures S1A and S1B) and anti-inflammatory properties make hBMSCs an ideal gene delivery vehicle [36]. It is imperative to demonstrate that genetic modification of hBMSCs do not affect their differentiation potential, stemness, immunological properties and secretion of paracrine factors. Adipogenic differentiation of transduced hBMSCs as confirmed by Oil Red staining demonstrated that ex vivo modification of hBMSCs with adenovirus did not alter their differentiation potential (Figure 2B). However, we did observe reduction in the extent of osteogenic differentiation of transduced hBMSCs. This reduction in osteogenic differentiation of transduced hBMSCs could be attributed to paracrine effects of accumulated hVEGF and hIL-1Ra in the differentiation media. These results are different from previously published reports that genetic modification of MSCs did not affect their differentiation potential [37], [38] and requires consideration of the effect of accumulated paracrine factors on the differentiation of genetically modified hBMSCs. Tracy et al have also shown that adenovirus transduction of BMSCs did not adversely affect their immunological properties as well as expression of chemokines and chemokine receptors [39]. However, they did not determine the effect of genetic modification on stemness as measured by expression of Nanog and Oct4 genes which are the key transcription factors for maintaining pluripotency and self-renewal potential of undifferentiated embryonic stem cells [40], [41]. Forced expression of Nanog or Sox2 maintained the expansion and differentiation potential of MSCs [42]. Here, we have shown that transduction of hBMSCs with Adv-hVEGF-hIL-1Ra did not alter the expression of Nanog and Oct4 (Figure 2A). In addition, previously we have shown that transduction of hBMSCs did not alter their paracrine secretions [43]. Our results indicate that adenoviral transduction of hBMSCs do not alter their proliferation, differentiation potential, expression of surface markers, immunological properties and stemness. Therefore, hBMSCs have the potential to be used as gene delivery vehicles.

MSCs as cell therapy has demonstrated efficacy both in the treatment of type 1 diabetes and islet transplantation [44]–[47]. There are conflicting reports whether hBMSCs undergo transdifferentiation to insulin secreting β cells or hBMSCs prevent islet apoptosis through the secretion of growth factors. Ito et al. have shown that VEGF secretion by BMSCs improved islet revascularization and thereby islet transplantation [46]. hBMSCs secrete an array of angiogenesis growth factors which promote islet vascularization and islet growth [48]. However, basal secretion of VEGF (Figure 3A) and other growth factors by hBMSCs was not enough to support the revascularization of islet graft soon after transplantation. Similarly, secretion of IL-1Ra by MSCs alone could not abrogate IL-1β mediated inflammation. Therefore, lentiviral transduction of MSCs to overexpress IL-1Ra has been performed to enhance their anti-inflammatory potential [49].

In this study, we have shown that hBMSCs can be genetically modified with bipartite adenoviral vector to promote islet revascularization and prevent islet apoptosis. Considering the safety, transduction efficiency and duration of transgene expression, adenovirus was preferred over retrovirus and plasmid DNA. Ex vivo genetic modification of hBMSCs resulted in significant co-expression of hVEGF and hIL-1Ra (Figures 3 A & B). This pattern of transgene expression is desirable as islet apoptosis occurs immediately after transplantation.

hBMSCs and islets can be either co-cultured together or separated through a cell culture insert. We have co-cultured islet and hBMSCs together as reported previously by Duprez et al [50]. hBMSCs having special affinity for islets through chemokines adhere to islets (Figure S2) and this interaction is desirable as hBMSCs will inhibit islet inflammation and create local immune suppressive milieu. Co-culturing with genetically modified hBMSCs protected islets against inflammatory cytokines and improved the islet viability as evident from Calcein AM/PI staining (Figure 4).

To determine whether co-transplantation of human islets with Adv-hVEGF-hIL-1Ra transduced hBMSCs can improve the outcome of islet transplantation, mean blood glucose levels were measured in NSG diabetic mice after transplantation of islets alone or with hBMSCs before or after transduction with Adv-hVEGF-hIL-1Ra (Figures 7 and 8). Co-transplantation of islets with Adv-hVEGF-hIL-1Ra transduced hBMSCs significantly improved diabetic reversal ratio and response to intraperitoneal glucose challenge (Figures 6D and 8). hvWF factor staining as a marker of endothelial cell proliferation was more evident in Adv-hVEGF-hIL-1Ra transduced hBMSCs (Figure 10). Prominent green staining in Adv-hVEGF-hIL-1Ra transduced hBMSCs indicates that hVEGF gene expression enhanced the endothelial cell proliferation of transplanted islets. To correlate the advantage of islet revascularization with the islet mass in the kidney capsule, we also stained for human insulin. Cotransplantation of islets with Adv-hVEGF-hIL-1Ra transduced hBMSCs displayed significantly higher levels of insulin content than un-transduced hBMSCs or islets alone (Figure 9). These findings correlated well with serum human insulin and C-peptide levels (Figure 7) and immunofluorescent staining (Figures 9 & 10).

Our results and previous reports have shown that hBMSCs can be used as cell and gene therapy to improve the outcome of islet transplantation. However, there are several issues which still need to be resolved before hBMSCs can be used clinically. Cytogenetic stability of hBMSCs after viral transduction needs to be established to allay safety issues of malignant transformation. To improve the transduction efficiency of hBMSCs and thereby reduce the viral dose, we plan to insert RGD peptide sequence into the adenovirus fiber as we reported before [51]. Furthermore, efficacy studies of genetically modified hBMSCs in humanized mouse mice l would confirm protective action of hBMSCs against long-term graft rejection.

In conclusion, our results have demonstrated that hBMSCs can be used as gene delivery vehicles without affecting their stemness and differentiation potential. Over expression of VEGF and IL-1Ra protected islet viability, promoted revascularization and significantly improved the glycemic control in diabetic mice.

## Supporting Information

Figure S1 Human bone marrow derived mesenchymal stem cells (hBMSCs) prevent the proliferation and activation of alloreactive T cells. The percentage of successive generations of human T cells when stimulated with phytohaemagglutinin (PHA, 5 µg/mL) and allowed to proliferate for 2 days. T cells were labeled with carboxyfluorescein diacetate succinimidyl ester (CFSE) and subjected to analysis using flow cytometry. (A) Proliferation index was calculated based on successive generation of T cells. Data are presented as the mean ± SD (n = 3). (B) Representative flow cytogram Left, T cells alone; middle, T cells with PHA; right, T cells with hBMSCs.
(TIF)

Figure S2 Adherence and spreading of hBMSCs on human islets. hBMSCs were labeled with Qdots, and incubated with islets overnight at 37°C. Adherence and spreading of hBMSCs on islet surface was confirmed by fluorescent microscopy. (A) Bright field image (B) Fluorescent image (C) Merged image.
(TIF)
