# Supplementary figures and images for: Genetically Modified Human Bone Marrow Derived Mesenchymal Stem Cells for Improving the Outcome of Human Islet Transplantation
Source: PLoS One. 2013 Oct 29;8(10):e77591. doi: 10.1371/journal.pone.0077591 (PMC3812220; doi:10.1371/journal.pone.0077591)

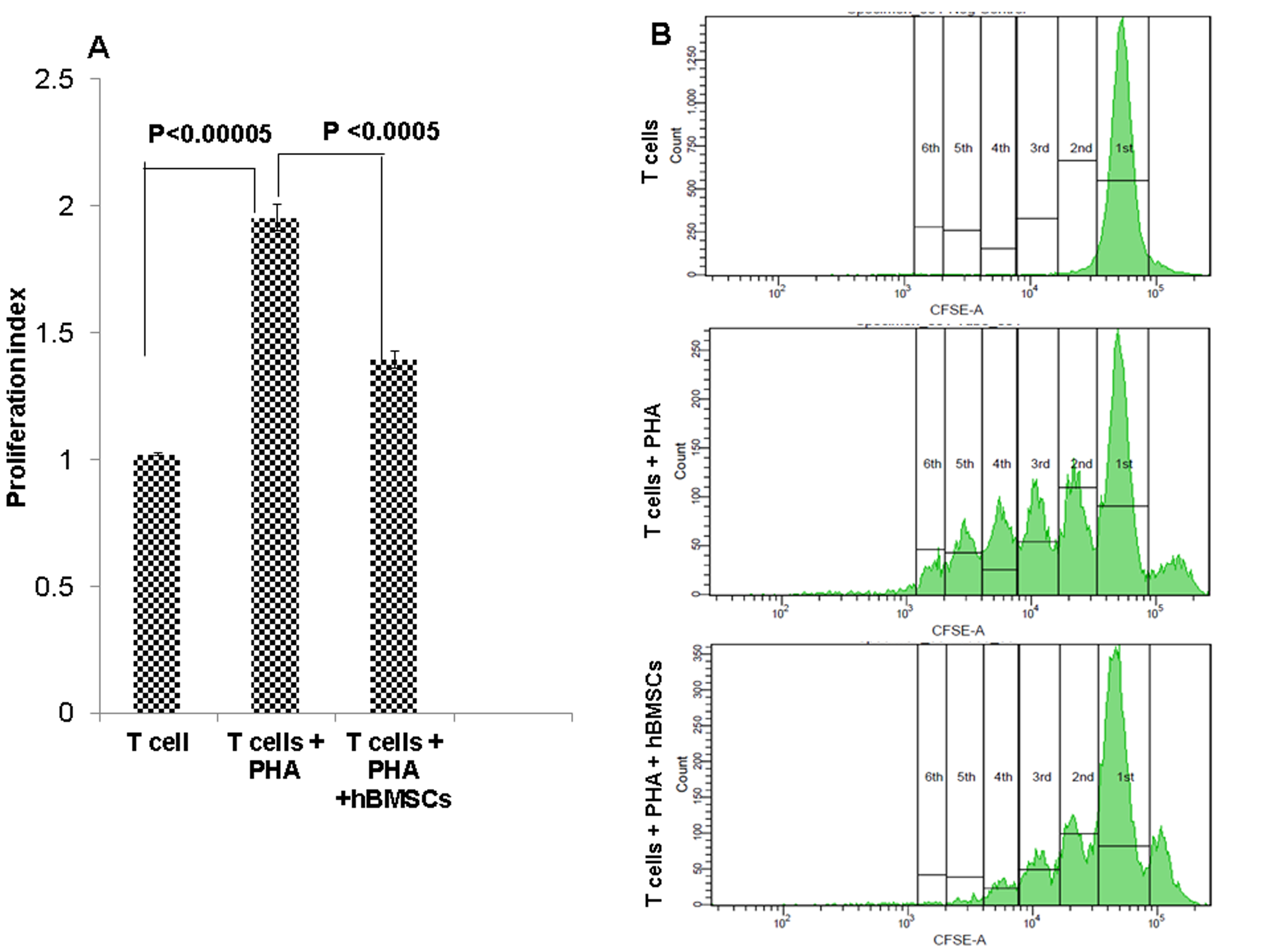

Supplement: Figure S1 — Human bone marrow derived mesenchymal stem cells (hBMSCs) prevent the proliferation and activation of alloreactive T cells. The percentage of successive generations of human T cells when stimulated with phytohaemagglutinin (PHA, 5 µg/mL) and allowed to proliferate for 2 days. T cells were labeled with carboxyfluorescein diacetate succinimidyl ester (CFSE) and subjected to analysis using flow cytometry. (A) Proliferation index was calculated based on successive generation of T cells. Data are presented as the mean ± SD (n = 3). (B) Representative flow cytogram Left, T cells alone; middle, T cells with PHA; right, T cells with hBMSCs. (TIF) [file pone.0077591.s001.tif]

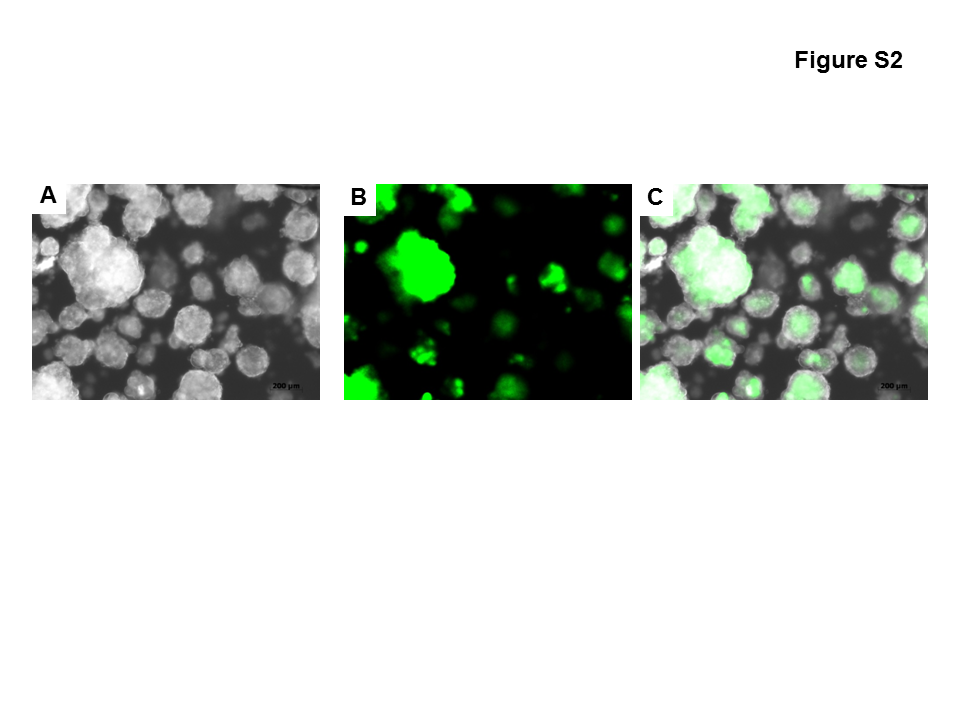

Supplement: Figure S2 — Adherence and spreading of hBMSCs on human islets. hBMSCs were labeled with Qdots, and incubated with islets overnight at 37°C. Adherence and spreading of hBMSCs on islet surface was confirmed by fluorescent microscopy. (A) Bright field image (B) Fluorescent image (C) Merged image. (TIF) [file pone.0077591.s002.tif]
